# Automated EMG-Based Classification of Upper Extremity Motor Impairment Levels in Subacute Stroke

**DOI:** 10.3390/s25226829

**Published:** 2025-11-07

**Authors:** Alexey Anastasiev, Hideki Kadone, Aiki Marushima, Hiroki Watanabe, Alexander Zaboronok, Shinya Watanabe, Akira Matsumura, Kenji Suzuki, Yuji Matsumaru, Hiroyuki Nishiyama, Eiichi Ishikawa

**Affiliations:** 1 Department of Neurosurgery, University of Tsukuba Hospital, University of Tsukuba, 2-1-1 Amakubo, Tsukuba 305-8576, Ibaraki, Japan; anastasiev.alexey.gb@u.tsukuba.ac.jp; 2 Center for Cybernics Research (CCR), Institute of Medicine, University of Tsukuba, 1-1-1 Tennodai, Tsukuba 305-8575, Ibaraki, Japan; 3 Department of Neurosurgery, Institute of Medicine, University of Tsukuba, 1-1-1 Tennodai, Tsukuba 305-8575, Ibaraki, Japan; aiki.marushima@md.tsukuba.ac.jp (A.M.); watanabe.hiroki.gb@u.tsukuba.ac.jp (H.W.); a.zaboronok@md.tsukuba.ac.jp (A.Z.); shinya-watanabey@md.tsukuba.ac.jp (S.W.); yujimatsumaru@md.tsukuba.ac.jp (Y.M.); e-ishikawa@md.tsukuba.ac.jp (E.I.); 4 Ichihara Hospital, 3681 Ozone, Tsukuba 300-3295, Ibaraki, Japan; matsumura.akira.ft@alumni.tsukuba.ac.jp; 5 Artificial Intelligence Laboratory, Center for Cybernics Research, Institute of Systems and Information Engineering, University of Tsukuba, 1-1-1 Tennodai, Tsukuba 305-8573, Ibaraki, Japan; kenji@ieee.org; 6 Center for Cyber Medicine Research, University of Tsukuba, 1-1-1 Amakubo, Tsukuba 305-8575, Ibaraki, Japan; nishiuro@md.tsukuba.ac.jp

**Keywords:** stroke, upper extremity, hemiparesis, motor assessment, outcome assessment, automated assessment, machine learning, supervised learning, electromyography (EMG)

## Abstract

Rehabilitation of upper extremity (UE) impairments after stroke requires regular evaluation, with standard methods typically being time–consuming and relying heavily on manual assessment by therapists. In our study, we propose automating these assessments using electromyography (EMG) as a core indicator of muscle activity, correlating passive and active EMG signals with clinical motor impairment scores. UE motor function in 25 patients was evaluated using the Fugl–Meyer Assessment for UE (FMA–UE), the Modified Ashworth Scale (MAS), and the Brunnstrom Recovery Stages (BRS). EMG data were processed via feature extraction and linear discriminant analysis (LDA), with 10-fold cross–validation for binary classification based on clinical score thresholds. The LDA classifier accurately distinguished impairment categories, achieving area under the receiver operating characteristic curve (AUC–ROC) scores of 0.897 ± 0.272 for FMA–UE > 33, 0.981 ± 0.103 for FMA–UE > 44, 0.890 ± 0.262 for MAS > 0, 0.968 ± 0.130 for BRS > 3, and 0.987 ± 0.085 for BRS > 4. Notably, resting–state EMG alone yielded comparable classification performance. These findings demonstrate that EMG–driven assessments can reliably classify motor impairment levels, offering a pathway to objective clinical scoring that can streamline rehabilitation workflows, reduce therapists’ manual burden, and prioritize patient recovery over assessment procedures.

## 1. Introduction

Stroke remains a major cause of long–term adult disability worldwide, with estimates suggesting nearly 12 million new cases annually [1]. Furthermore, the lifetime risk of stroke for individuals over 25 years old is estimated at 1 in 4, underscoring its widespread impact on people, healthcare systems, and communities [1,2,3]. A frequent and debilitating post–stroke outcome is upper extremity (UE) motor impairment, which significantly hinders patients’ ability to perform activities of daily living (ADLs) and return to social life, necessitating timely rehabilitation and continuous monitoring [2,3].

In clinical practice, the cornerstone of UE functional assessment relies on standardized clinical scales administered by skilled therapists, such as occupational therapists (OTs) [3,4,5]. Though essential, these methods are labor–intensive, inherently subjective, and difficult to scale [4,6,7,8,9,10]. Currently, a growing strain on rehabilitation resources is exacerbating this healthcare challenge [1,11]. For instance, demand for OTs in the U.S. is projected to grow by 14% from 2024 to 2034, significantly outpacing the growth rate projected for all occupations [12]. Moreover, clinical guidelines often recommend intensive therapy [13] (p. 8), such as at least 45 min per required discipline daily (e.g., NHS UK) or over 3 h of combined therapy daily in dedicated inpatient rehabilitation facilities (e.g., U.S. IRF standards) [3,5]. However, achieving these targets is often hampered by staffing levels and systemic constraints [11,12,14]. Studies indicate that actual therapy time received during hospital stays can be significantly lower, and patient–to–therapist ratios may exceed optimal levels, potentially limiting functional gains [2,15,16]. This gap between recommended and actual rehabilitation delivery in clinical settings underscores the urgent need for technologies to automate functional assessments, freeing therapists’ time for treatment rather than routine evaluations [10,17,18,19,20,21,22,23,24,25,26,27,28].

For decades, manual clinical instruments such as the Fugl–Meyer Assessment (FMA), the Modified Ashworth Scale (MAS), and the Brunnstrom Recovery Stages (BRS) have been the cornerstones of post–stroke motor assessment [4,5,6,9,10,17,18,19,21,24,25,26,27,28,29]. Each of these tests has specific target domains; for instance, MAS evaluates muscle resistance to passive stretching [6], BRS evaluates the recovery stage based on the presence of spasticity and the quality of synergistic movements [9], while FMA–UE includes complex tasks involving reflexes and isolated joint movements, as well as assessments of sensory function, balance, and pain [29]. These subjective scales are valued for their comprehensive structure, established validity, and reasonable though imperfect inter–rater reliability in clinical practice [4,10]. Nevertheless, their reliability, while often satisfactory, is not absolute and leaves significant room for improvement. For example, Hernández et al. reported high item–level agreement (79% and higher) for the FMA–UE among different therapists [8], yet a meta–analysis by Meseguer–Henarejos et al. characterized the reliability of the MAS as satisfactory in certain comparisons [6]. Moreover, beyond the considerable time required for administration (e.g., over 20 min for the FMA–UE), the ordinal scoring systems of these assessments are often insensitive to small but clinically meaningful improvements, relying largely on an individual clinician’s visual observation [17,29]. This is particularly evident in the BRS, where the transition between stages (e.g., from BRS 3 to 4) is a gradual, dynamic process rather than a discrete event [4,9]. Such ambiguity can lead to differing interpretations of what constitutes “minimal” versus “clear” voluntary control. This practical ceiling on assessment depth is a key driver for developing objective, automated methods derived from patients’ biosignal processing [17].

To address these conventional limitations, reduce inter–rater variability, and improve recovery outcomes, recent research has increasingly focused on the utilization of biosignal processing [24,25,26]. Notably, electromyography (EMG) provides direct insight into motor ability, as myoelectrical signals directly reflect muscle activation [10]. Inertial measurement units (IMUs) offer complementary kinematic data, and the integration of these two biosignals has emerged as a leading approach for objective motor assessment [17,18]. In the scope of clinical practice, by capturing EMG activity with or without movement kinematics, these patient data, when coupled with machine learning (ML), feature engineering, or related computer–based processing, can offer insights into motor unit recruitment and activation patterns [20,21,22,23,24,30,31,32,33]. Such data are important for understanding stroke–related deficits and tracking patient recovery, as the obtained biosignals may reflect the degree of motor impairment and correlate with scores assessed by OTs [10,23]. For illustration, Meng et al. achieved high classification accuracy for BRS (late recovery stages) using ML models applied to data from five wearable surface EMG sensors and multi–axis IMUs collected from stroke survivors performing ADLs, where linear discriminant analysis (LDA) and support vector machine (SVM) classifiers both reached 96.3% subject–level accuracy [30]. Moreover, Zhou et al. showed that ML methods applied to IMU bracelet–type data input have enabled precise prediction of FMA–UE subscale scores in post–stroke patients [33]. Similar prediction scores have been reported in multimodal EMG/IMU data fusion for UE motor evaluation [22].

Alternatively, for patients unable to perform movements correctly (i.e., BRS below 3), EMG or IMU analysis can detect subtle muscle activation gaps, micro–contractions, or abnormal coactivations that are not visible but traceable via signal recordings, enabling recognition of paretic movements [18,19,20,28]. Likewise, previous research shows that canonical supervised learning classifiers or neural networks can accurately classify ADL–related hand movements using only EMG signals from the affected side or through bilateral data fusion [10,31,32]. Moreover, interpretable ML frameworks analyzing EMG patterns in chronic stroke patients have also distinguished impaired myoelectric activity, correlating with clinical motor assessments [21,24]. However, such tools for acute and subacute patients remain limited due to both the patients’ physical conditions and technical constraints [31].

Despite progress in studies on objective biosignal–driven motor assessments, states with mild or absent movements are often overlooked, as current research primarily focuses on the post–discharge period [10,18]. In contrast, research on the subacute period of stroke remains limited, as patients in this stage are often mentally and physically weak and unable to control their extremities under hemiplegic conditions. This significantly hinders kinematic data collection and analysis (i.e., reducing ML performance) and shifts focus to complex, non–scalable multimodal applications, such as combined evaluation of EMG and electroencephalography (EEG) [17]. Moreover, most existing systems with fused signal–processing modalities (e.g., EMG/IMU–based arm training, where assessments are performed in parallel with recovery sessions) are stationary, non–portable and require medical assistance [10,17,22]. However, the development of portable and scalable applications, featuring EMG–based scoring models that integrate into UE motor scales, which can be applied in in–hospital populations, has not been fully formalized [18]. Finally, current methods are typically tuned for single–scale assessment, limiting multi–scale automated evaluations [21,22,24,25,30]. This is critical, as multiple scales are needed for more precise and detailed comprehension of recovery trajectories, which nowadays can only be obtained through time–consuming manual procedures [13,14,15].

In this work, based on data collected from 25 acute and subacute stroke patients, we present an automated approach to classify the FMA–UE, BRS, and MAS stroke motor assessments, using only four–channel EMG from resting states and hand gestures related to ADLs. The justification of the need for this automated assessment approach is to provide a supportive tool for therapists. It aims to automate time–intensive aspects of manual stroke assessment, thereby alleviating the burden caused by the shortage of OTs in rehabilitation units and promoting individualized analysis of patient recovery trajectories [10,16]. Our novel pipeline incorporates paretic EMG data from subacute stroke patients with weakened or absent movements and relies on a low–channel EMG system with binary feature extraction for each motor scale to classify motor impairment levels via LDA, thereby enabling new applications for wearable electronics in precision medicine.

## 2. Materials and Methods

### 2.1. Clinical Experiment Design Settings

This study re–analyzed a rare and unique dataset of EMG signals collected from acute and subacute stroke patients with UL paresis, originally recorded in our previous study, alongside manual motor impairment assessments [28,31,32]. Detailed patient demographics and collected clinical data are presented in Section 3.1.

Patients were recruited from the University of Tsukuba Hospital, Japan, with inclusion criteria including age over 18 years, a confirmed cerebrovascular accident (cerebral infarction or intracranial hemorrhage) within six weeks post–onset, UE motor deficits, and the ability to understand the observational procedure, while exclusion criteria included severe cognitive impairment or inability to provide informed consent.

EMG signals were recorded using a portable, wireless 8–channel EMG device (PLUX Wireless Biosignals S.A., Lisbon, Portugal; model 8CH HUB 19022021), equipped with a 16–bit analog–to–digital converter and a sampling rate of 1 kHz. Four Ag/AgCl hydrogel electrodes were placed on the main forearm flexors, extensors, and thenar area. During the recordings, participants performed on the affected side a set of simple hand gesture attempts reflecting ADLs, including rest, hand fist, index pinch, wrist flexion, extension, palm opening, and upright thumb [30,31]. Each gesture was attempted around 15 times per session, starting from an idle posture. Experimental sessions and motor assessments lasted 30–60 min and were conducted in a hospital setting with the assistance of skilled therapists. A visualization of the overall data–acquiring process for each gesture is presented in our previous study on empirical distal movement decoding in post–stroke patients [32].

**Figure 1:**
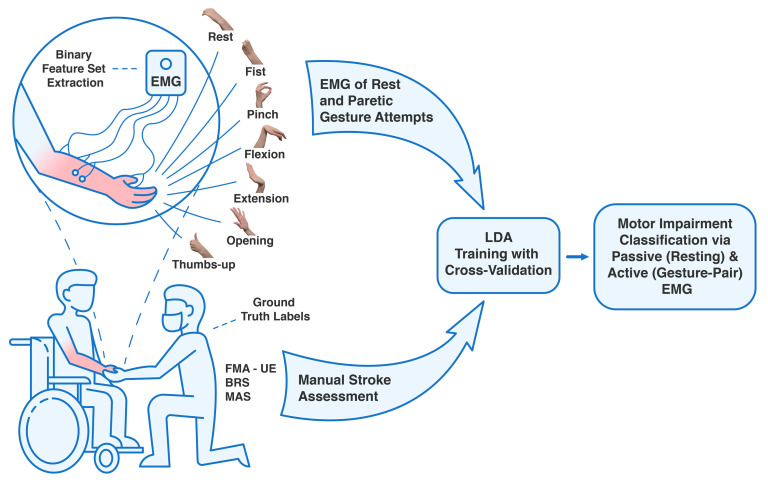
A machine learning workflow for classifying motor impairment levels using EMG signals in subacute stroke patients. Forearm and hand surface EMG data were acquired from the hemiplegic side of participants during the resting state and six distinct volitional gesture attempts. Manual motor assessments (FMA–UE, BRS, and MAS) were conducted by a therapist, and the resulting scores served as the ground truth labels for classification. Binary feature sets were extracted from the EMG data to train an LDA classifier for two separate models: a passive prediction (using only resting EMG) and an active prediction (using data from a gesture–pair). The model’s classification performance was evaluated using 10-fold cross–validation.

For each patient, motor impairment was assessed using FMA–UE (with scores from 0 to 66), BRS (stages 1 to 6) for the UE and forearm, and hand, and MAS (0 to 4) for the elbow, wrist, and hand [4,5,6,29,33]. Given the sample size and patients’ characteristics, to ensure better validity of the results utilizing EMG signals of hand movements, only the hand scoring components of the BRS and MAS were used for signal preprocessing and binary classification. An illustration summarizing the following observational clinical research can be found in Figure 1.

### 2.2. Data Preprocessing

Raw EMG signals were preprocessed and combined into a single dataset to ensure quality and consistency for binary classification. First, the signals were converted to microvolts using the device’s transfer function (±1.5 mV range). Next, a fourth–order Butterworth bandpass filter (20–300 Hz) was applied to reduce noise and motion artifacts [31]. The signals were then demeaned, followed by full–wave rectification and normalization via root–mean–square envelope calculation to account for amplitude variations across patients [24,34].

Gesture segments (including attempted gestures) were automatically detected using a centered moving average for peak identification, followed by manual validation to ensure correct muscle signatures corresponding to movements or rest [32]. At a sampling rate of 1 kHz, each gesture attempt yielded 750 samples. A detailed depiction of myoelectric gesture signatures on the affected side can be found in our recent publication [28].

### 2.3. Feature Extraction

A feature extraction from preprocessed signal segments was implemented to represent hand movements and rest states as EMG time series, which then served as input for predicting stroke motor impairment scales [18,24,30]. Unlike our previous experiments, no gesture recognition methods were applied.

Here, to enable real–time application and account for the unique characteristics of each scale, such as the FMA–UE (encompassing motor and sensory items) and the MAS (assessing muscle tone), four EMG features were selected: two for passive evaluation during rest and two for active assessment of volitional hand movements. To enable objective mapping of EMG signals to motor impairment scores, a targeted set of features was extracted from the preprocessed segments [32]. The selection was guided by the semi–brute force navigated amalgamation in linkage of EMG features strategy, and the resulting paired–feature vectors were extracted for each gesture attempt or idle state [28]. After normalization, all EMG sources were transformed into Euclidean–space feature vectors to prepare the time–series data for supervised learning [31,35].

#### 2.3.1. EMG Features for FMA–UE Categorization

Given that the FMA–UE is a multifactorial scale with 66 points across different domains, two time–domain EMG features were selected for the evaluation: the mean absolute value slope (MAVS) and the log of the simple square integral (LSSI) [32,35]. These features were chosen for their robustness to noise and established correlation with applied muscle force across the four EMG channels, effectively reflecting the force exerted during tasks relevant to FMA [28].

For the passive EMG assessment, the 3rd–order linear predictive coefficient (LPC3) and the energy of wavelet packet (EWP) coefficients were used [32]. These features were chosen to account for abnormal muscle tone and non–volitional activity. LPC3 specifies the EMG signal’s spectral shape and significantly reduces noise, while EWP quantifies the power within specific frequency bands, making them suitable for interpreting the FMA–UE from resting–state EMG [28].

#### 2.3.2. EMG Features for BRS Categorization

Given the complexity of tracking BRS recovery, distinct binary feature sets were selected to classify thresholds of BRS greater than 3 and BRS greater than 4.

For the BRS greater than 3 binary classification thresholds, the features were chosen to detect the initial emergence of voluntary control. For active hand movement attempts, the average amplitude value (AAV) and a 4–bin frequency histogram (FTHT4) were used to capture basic muscle output and its general spectral distribution [28]. For the corresponding passive state, a handcrafted modified mean absolute value (MMAV3) was combined with a second–order root–mean–square value (RMSV2) [35]. The MMAV3 applies a weighting window that emphasizes the initial portion of the EMG signal [32]. This design is crucial for assessing the start of muscle bursts, as patients at this stage may be able to initiate a volitional movement but cannot sustain it for a prolonged period [10].

For BRS greater than 4, the features were selected to reflect the gradually improving quality and isolation of movements [4,9]. For active movements, enhanced wavelength (EWL) and a 50th percentile amplitude feature (PERC2) were used [28]. These features are more sensitive to the increasing complexity and stability of the EMG signal as patients progress, and muscle control adapts [28]. For the passive state at this stage, the power spectrum ratio (PSR) and median energy of the wavelet packet (MEWP) were used to quantify the evolving characteristics of the resting muscle tone [10,28].

#### 2.3.3. EMG Features for MAS Categorization

For the relation between hand movements and MAS, the modified mean absolute value (MMAV) and scaled mean absolute value (SMAV) were used in the active EMG assessment, as they provide a robust representation of volitional muscle effort in the presence of spasticity [28]. MMAV offers a stable measure of sustained contraction by emphasizing the middle portion of muscle activation, making it less susceptible to noise at the onset and offset of movement, whereas SMAV normalizes the movement signal by its duration.

For MAS stroke assessment in the rest state, the selected features were used to quantify spasticity, characterized by hyperexcitable, involuntary muscle activity and abnormal co–contractions [4]. Wavelet packet entropy (WENT) was employed to capture the complexity and predictability of the EMG signal’s time–frequency signature [28]. Concurrently, correlation coefficients of square–root–normalized values (CC–S) provided a measure of abnormal synergies between different muscle locations [28,32].

### 2.4. Supervised Learning and Model Validation

Given the limited sample sizes in stroke research conducted in clinical settings, a binary classification approach was used to evaluate the ability of supervised learning to classify motor impairment levels from stroke–related myoelectrical signatures. This involved classifying patient data as being either above or below a clinically significant threshold for each motor impairment scale.

The following clinically relevant thresholds were used to create the binary classification tasks:

- FMA–UE: greater than 44 (i.e., mild vs. moderate–to–severe impairment) and greater than 29 (i.e., non–severe vs. severe impairment) [29,36];
- BRS: greater than 3 and 4 (to assess progression through recovery endpoints [4];
- MAS: greater than 0 (presence vs. absence of clinically significant spasticity) [6,7].

An LDA classifier was used for all classification tasks. This supervised algorithm was chosen for its computational efficiency and demonstrated effectiveness in handling EMG feature vectors, particularly in applications with a limited number of features, where it is less prone to overfitting compared to more complex models [10,30,32].

To ensure robustness and generalizability, we implemented 10-fold cross–validation [28]. Each iteration used all gesture data from patients (including rest), with subject–wise partitioning to preserve patient independence. The dataset (*n* = 25) was randomly split 100 times, where in each iteration, data from 23 patients were allocated for training and data from 2 patients for testing [31]. Final performance metrics for each classification task were reported as the mean score across all 100 iterations. Ground–truth labels were derived from therapist–administered FMA–UE, MAS, and BRS assessments conducted immediately after EMG acquisition.

In the single–gesture evaluation, one volitional gesture (e.g., hand fist or the rest–state EMG signal segment) was used to predict clinical thresholds. In the combined–gesture evaluation, pairs of gestures (e.g., index pinch and hand opening) were analyzed, providing a broader assessment of whether aggregated myoelectrical signatures could predict UL impairment levels.

### 2.5. Performance Metrics

Model performance for each binary classification task was evaluated using a comprehensive set of metrics: accuracy (ACC), recall (REC), precision (PREC), specificity (SPEC), F1–score (F1), the area under the receiver operating characteristic curve (AUC–ROC), and the area under the precision–recall curve (AUC–PRC) [24,31,37].

AUC–ROC and AUC–PRC were prioritized as key metrics because they are particularly effective for evaluating classifier performance on imbalanced clinical datasets [31,38]. AUC–ROC provides a threshold–independent measure of a model’s overall discriminative ability, whereas AUC–PRC is more sensitive to performance on the positive class, which is critical when identifying patients who have crossed a specific impairment threshold [39]. Confusion matrices were generated for binary–gesture models (see Figure S1).

### 2.6. Statistical Analysis

To determine the predictive power of the EMG–based automated stroke motor assessments, the mean accuracies of the single–gesture model (7 gesture label classes) and the binary–gesture model (21 possible gesture label combinations), obtained from 100 iterations of 10-fold cross–validation via LDA, were independently compared for each scale. The non–parametric Mann–Whitney U test was used to assess the statistical significance of differences between the mean accuracies of any two models within these comparisons (e.g., comparing the ‘resting EMG’ model versus the ‘hand fist’ model) [40]. This test was selected because the accuracy values from 100 iterations could not be assumed to follow a normal distribution. The null hypothesis was rejected at significance levels of *p* < 0.05, *p* < 0.01, and *p* < 0.001.

All feature extraction was performed in MATLAB (R2025a, The MathWorks, Inc., Natick, MA, USA) on a Windows 11 equipped with an Intel^®^ Core™ i9–7900X CPU (Intel Corporation, Santa Clara, CA, USA) and 128 GB of RAM. The ML models were implemented in Python 3.12.3 (using the NumPy 2.1.3, Pandas 2.3.1, SciPy 1.16.0, Scikit-learn 1.7.0, Matplotlib 3.10.3, and Seaborn 0.13.2 libraries) on an Ubuntu 24.04 system with a 14th Gen Intel^®^ Core™ i5–14500 processor, 64 GB of RAM, and an NVIDIA^®^ RTX A2000 GPU (NVIDIA Corporation, Santa Clara, CA, USA).

## 3. Results

The following section presents the performance of the LDA classifier in predicting the FMA–UE, BRS, and MAS scores. The results are organized into three categories: Section 3.1 describes the demographics of the study patients, Section 3.2 reports the passive assessment based on resting–state EMG, and Section 3.3 presents the active assessment based on EMG signals from volitional hand movements in a combinational analysis of all possible gesture label classes.

### 3.1. Patient Demographics and Clinical Characteristics

The observational study cohort consisted of 25 participants with confirmed post–stroke hemiparesis. The demographic and clinical characteristics of the participants are summarized in Table 1.

### 3.2. Passive EMG Assessment for the Motor Impairment Prediction

**Figure 2:**
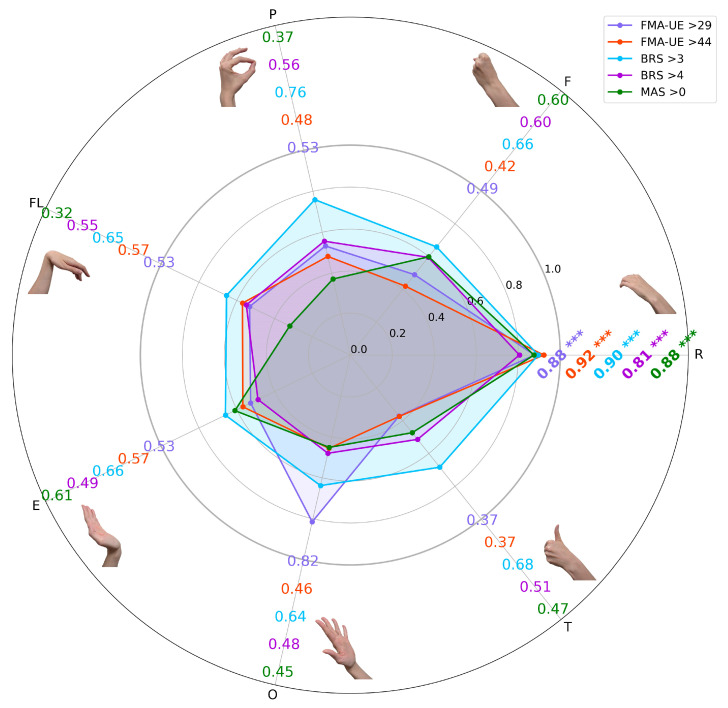
The radial chart of binary classification accuracy for single EMG gesture signals across five clinical thresholds (FMA–UE > 29, FMA–UE > 44, BRS > 3, BRS > 4, MAS > 0). Each colored contour represents the mean LDA accuracy for a single gesture per threshold based on 100 iterations of 10-fold cross–validation, with bold values indicating the best–performing gesture label for each category. Asterisks represent statistically significant performance differences compared to the best class within a single clinical threshold (*p* < 0.001, Mann–Whitney U test). The outer radial axis of gesture labels corresponds to the EMG gesture signals used for LDA classification. Abbreviations: R, rest; F, hand fist; P, index pinch; FL, wrist flexion; E, wrist extension; O, hand opening; T, thumbs–up.

The predictive performance of LDA based on EMG features for classifying clinical impairment thresholds is presented in Figure 2.

A primary finding across all evaluated scales (FMA–UE, BRS, and MAS) was the superior performance of classification derived from the passive rest state myoelectrical data. In every classification task, the resting–state EMG features yielded a mean accuracy of over 0.80, which was significantly higher than the accuracies achieved using features from any of the active volitional gestures (*p* < 0.001 for all comparisons).

The highest overall performance was achieved in the classification of the FMA–UE > 44 threshold, which reached a mean accuracy of 0.92 using the LPC3 and EWP features from the rest state. A similarly high accuracy of 0.90 was obtained for the BRS > 3 threshold, using the MMAV3 and RMSV2. A comprehensive summary of all performance metrics (e.g., F1–score) for each task is provided in the Supplementary Materials (see Table S1).

**Figure 3:**
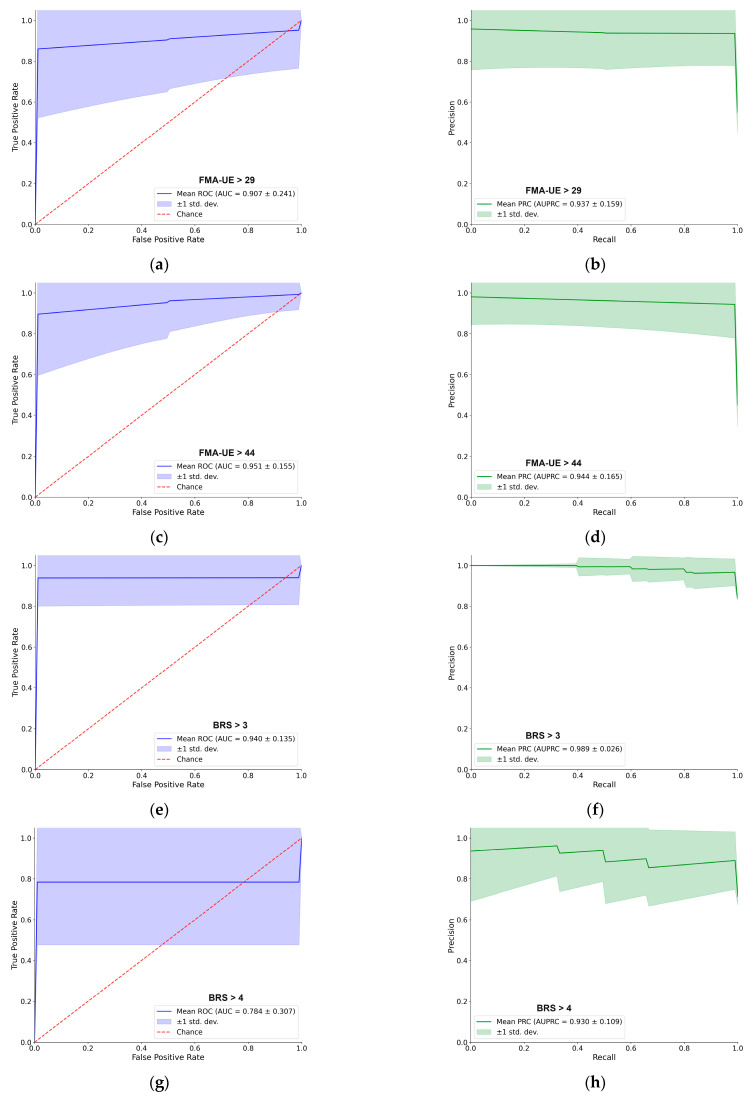

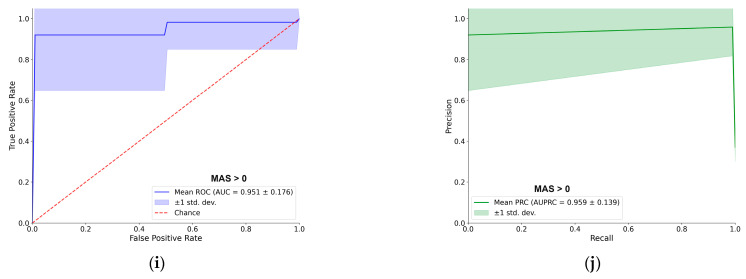
Performance of the LDA classifier using only resting–state EMG. The figure shows the receiver operating characteristic (AUC–ROC) curves (left column, blue) and precision–recall curves (AUC–PRC) (right column, green) for five different clinical thresholds. The solid line represents the mean performance, and the shaded area indicates the ±1 standard deviation over 100 cross–validation iterations. From top to bottom, the rows correspond to the classification of: (**a**,**b**) FMA–UE > 29, (**c**,**d**) FMA–UE > 44, (**e**,**f**) BRS > 3, (**g**,**h**) BRS > 4, and (**i**,**j**) MAS > 0.

For better navigation, Figure 3 provides a detailed performance evaluation of the LDA classifier for the passive (resting–state) EMG assessment. For each of the five clinical thresholds, the figure presents both the ROC and PRC. Overall, the LDA demonstrated excellent predictive capacity. For most tasks, the AUC–ROC was approximately 0.90 or higher, and the AUC–PRC was around 0.95. A notable exception was the BRS > 4 classification, which yielded a lower AUC–ROC of 0.78, although the AUC–PRC remained high at 0.93. The shape of the PRC for this specific task also indicates some instability in precision across different recall levels. Each curve represents the mean performance across 100 cross–validation iterations, with the shaded region indicating the ±1 standard deviation, providing a clear visualization of the consistency of supervised models [31].

### 3.3. Active Movement EMG Interpretation into Stroke Impairment Motor Assessments

**Figure 4:**
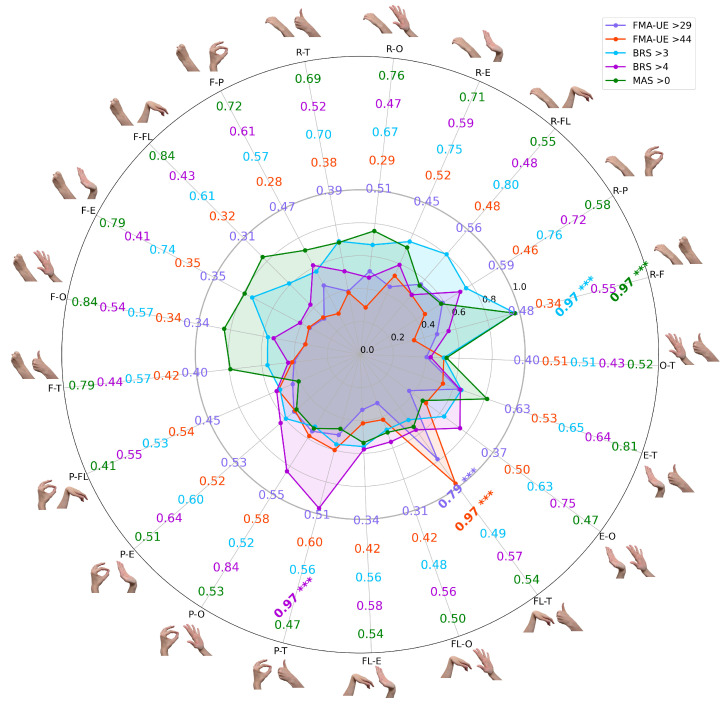
The radial chart of binary classification accuracy for EMG signals using all possible two–gesture combinations across five clinical thresholds (FMA–UE > 29, FMA–UE > 44, BRS > 3, BRS > 4, MAS > 0). Each colored contour shows the mean LDA accuracy per threshold based on 100 iterations of 10-fold cross–validation, where each axis corresponds to a specific binary gesture pair. Bold values indicate the best–performing binary combination for each clinical category, and asterisks represent statistically significant differences in mean accuracy compared to the best–performing combination within the same group (*p* < 0.001, Mann–Whitney U test). Abbreviations: R, rest; F, hand fist; P, index pinch; FL, wrist flexion; E, wrist extension; O, hand opening; T, thumbs–up.

To determine if combining myoelectrical signatures from different tasks could improve predictive power, all possible binary gesture combinations were evaluated. The classification performance for each clinical threshold is presented in Figure 4. For a more detailed visualization of the classification results, based on confusion matrices, please refer to Figure S1 and Table S2.

These bar charts illustrate the total mean LDA classification accuracies across all possible two–task combinations of gestures, with error bars indicating standard deviation from 100 iterations. Each bar represents the performance of a model trained on two distinct gesture labels (e.g., R–F combines rest and hand fist). All combinations showed statistically significant predictive power (*p* < 0.001), underscoring the potential of minimal EMG data for objective, multi–scale assessments in clinical stroke rehabilitation.

The results revealed unique patterns with statistical significance (*p* < 0.001) across comparisons, where the highest accuracies reached 0.97 for most thresholds (FMA–UE > 44, BRS > 3, BRS > 4, and MAS > 0), while the maximum for FMA–UE > 29 was 0.79. Notably, for thresholds like BRS > 3 and MAS > 0, a dominant gesture combination emerged, showing significant differences when including a binary combination with the resting EMG gesture class; for example, R–F (rest and fist) was the top performer across these thresholds. Interestingly, across different scales and extracted features (under cross–validation settings), thumbs–up EMG signatures consistently contributed to high accuracy, except for the BRS > 3 classification settings. For illustration, in FMA–UE > 29, lower overall accuracies were observed, but predictive power remained significant (*p* < 0.001), with thumbs–up gesture labels enhancing results. In FMA–UE > 44, strong performance distinguished mild from moderate–to–severe impairment, specifically boosted by thumbs–up hand movement. For BRS > 3, the approach effectively detected early voluntary control emergence, whereas thumbs–up labels had a negative impact on the model’s binary classification. BRS > 4 highlighted improving movement isolation, with high accuracy from thumbs–up and similar gestures. Finally, MAS > 0 proved robust in identifying spasticity, emphasizing sustained contractions in volitional attempts, with multiple combinations exceeding 0.75.

**Figure 5:**
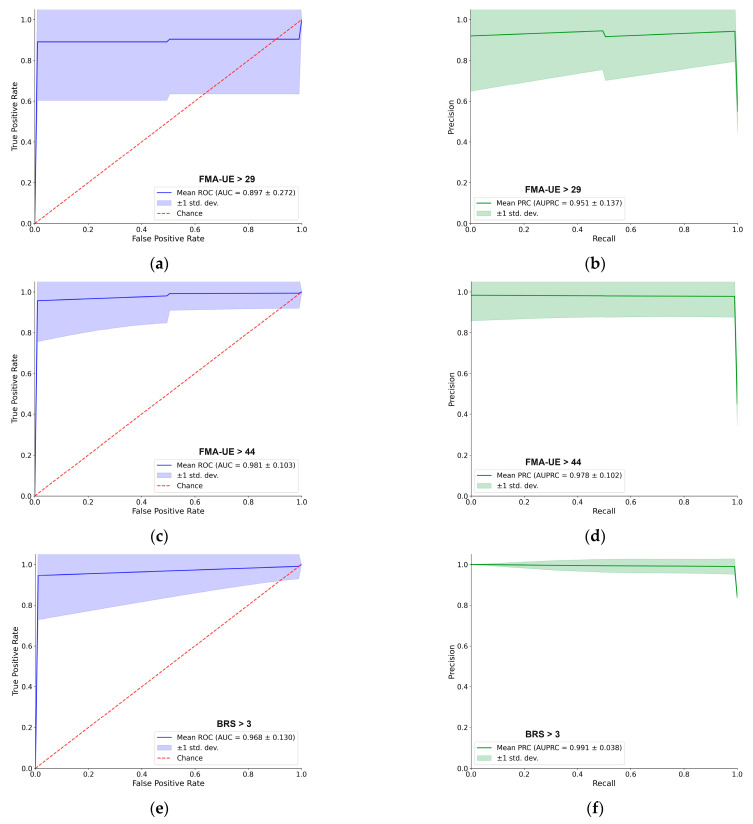

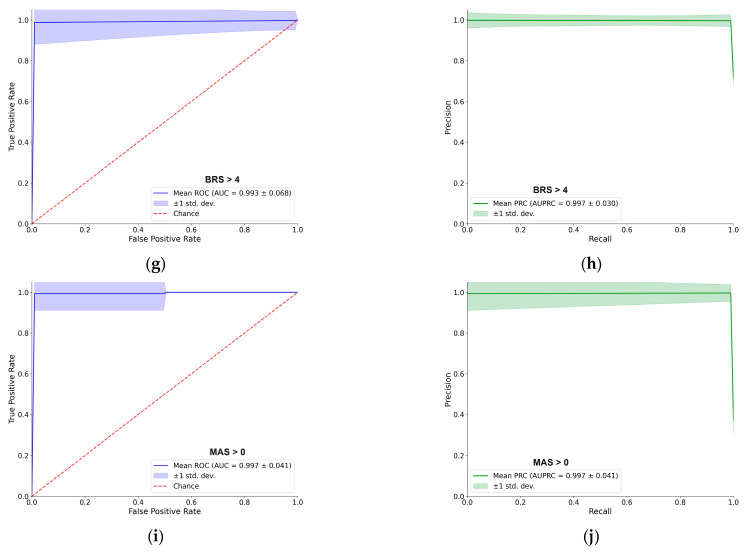
Performance of the LDA classifier for the best binary combination of gesture–label EMG signals. The figure shows the AUC–ROC curves (left column, blue) and AUC–PRC (right column, green) for five different clinical thresholds. The solid line represents the mean performance, and the shaded area indicates the ±1 standard deviation over 100 cross–validation iterations. From top to bottom, the rows correspond to the classification of: (**a**,**b**) FMA–UE > 29, (**c**,**d**) FMA–UE > 44, (**e**,**f**) BRS > 3, (**g**,**h**) BRS > 4, and (**i**,**j**) MAS > 0.

Two consistent patterns emerged across the different classification tasks. First, combinations that included the rest gesture label were frequently among the highest–performing models, possibly confirming the rich diagnostic value of resting–state EMG [41]. Second, the thumbs–up gesture, tested on different scales with different features, when paired with other movements, consistently contributed to high–accuracy models, particularly for thresholds other than BRS > 3, suggesting its unique myoelectrical signature is a strong indicator of advancing motor recovery. ROC and PRC curves for the best–performing binary combinations of paretic hand gesture attempts were visualized in Figure 5.

A score comparison of the single–task and two–task models reveals the benefit of combining myoelectrical signatures of gestures. As shown in Figure 4, using a two–gesture input generally improved the ML performance metrics compared to models based on a single gesture (see Figure 2).

Moreover, the binary–task approach not only improved overall performance but also reduced the variance in both the ROC and PRC curves, indicating a more stable and reliable binary prediction. While the overall shapes of the curves are similar to those from the single–task rest assessment, the improved stability and higher AUC values demonstrate the advantage of the two–task input. The specific best–performing binary combinations for each clinical scale are detailed in Figure 5.

## 4. Discussion

In a clinical sense, the motor assessment made by physicians of post–stroke survivors can be presented in a set of patients’ EMG signal time series (i.e., muscle group contractions) [10,41]. Here, feature extraction and supervised machine learning were used to build a model to predict the assessments based on the surface EMG output collected on the affected upper extremity in acute and subacute stroke patients.

This study demonstrates the significant potential of a streamlined, low–channel EMG system to provide an objective, accurate, and automated interpretation of post–stroke motor impairment in acute and subacute patients. We have shown that a carefully selected, minimal set of EMG features (i.e., two features per binary threshold), when processed by a simple LDA classifier, can predict scores across three fundamentally different clinical scales (FMA–UE, BRS, and MAS) with high fidelity (specifically, up to 0.97 accuracy for thresholds like FMA–UE > 44, BRS > 3, BRS > 4, and MAS > 0, and 0.79 for FMA–UE > 29), with statistical significance (*p* < 0.001) in all cases. These metrics from subacute stroke EMG data (*n* = 25) showed valid ROC and PRC curves with scores generally above 90% across most tests. Notably, gesture combinations incorporating rest and dynamic movements (e.g., rest and fist) emerged as dominant performers, underscoring the diagnostic value of resting–state EMG and volitional attempts like thumbs–up, which consistently enhanced accuracy except in early BRS stages [30]. In essence, combining EMG features from rest practically means using a single gesture for assessment, with rest as an additional EMG source for LDA, requiring patients to perform only one gesture (around 15 attempts in our settings). These patterns reveal traceable myoelectrical signatures that align with clinical domains: sustained contractions for spasticity (i.e., MAS assessment), initial bursts for voluntary control emergence (BRS > 3), and complex stability for higher recovery (BRS > 4 and FMA–UE > 44) [4,33].

Our most significant finding is the remarkable predictive power of the passive resting state. The myoelectrical signature of a patient’s paretic forearm at rest consistently served as the most robust indicator of overall motor function, often outperforming features derived from active, volitional movements [24,26]. This underscores that non–volitional, underlying muscle tone contains a useful, quantifiable biomarker for the overall state of the sensorimotor system after stroke [10,18]. Given the obtained scores in our study, we suggest that reliable assessments may be possible even in severely impaired patients unable to produce consistent voluntary movements. Moreover, since that recovery is a dynamic process, the ability to accurately and frequently track changes in UE motor function is paramount for guiding therapeutic interventions and maximizing patient–specific outcomes [3,4,9].

Furthermore, this work challenges the prevailing trend that complex, multi–modal sensor fusion (e.g., EMG+IMUs or EEG) is necessary for accurate assessment [17]. By achieving high classification accuracy with only four channels of surface EMG, we present a more practical and clinically feasible alternative [10,33]. This approach avoids the significant burden associated with systems requiring kinematic data from patients with IMU or EEG, making it particularly well–suited for the acute and subacute stages of recovery [17,18].

A key limitation is our reliance on binary classification rather than correlation or regression analysis, which could predict exact scores from myoelectric data. Unlike methods employing regression for continuous scoring (e.g., achieving R^2^ > 0.8 in chronic stroke via multi–feature EMG models), we initially categorize assessments into motor impairment levels (i.e., mild vs. severe deficit) [33,38]. This binary structure, while efficient for initial thresholding, can be iteratively refined to narrow score ranges; alternatively, incorporating more data, EMG features, and patient samples could enable regression, which we plan to test in future research [28,33,38]. Furthermore, pursuing more finite questionnaire scoring through manual assessments is impractical, as it imposes an unrealistic time and workload burden on clinicians and patients.

An additional limiting aspect is that the clinical research was performed on a relatively small cohort of 25 stroke patients. Although a significant number of EMG signatures of gestures (approximately 15 per participant) were recorded, the limited sample size may restrict generalizability. Nonetheless, the use of 10-fold cross–validation with 100 iterations, as commonly applied in similar studies, was employed to enhance the robustness and consistency of the results [28,42]. For greater validity of further predictive models, a train–validation–test split will be employed in subsequent experiments to avoid biases in ML classification pipelines [43].

## 5. Conclusions

The main outcomes of this study can be summarized in the following three key findings. First, a low–channel system alone was sufficient for the successful assessment of stroke–related EMG signal data. Second, only two extracted features were required for each LDA–based binary threshold decoding, demonstrating the potential suitability of this approach for real–time clinical applications [3,14]. Finally, the study revealed that even a single–movement hand gesture EMG recording or, most importantly, resting–signal EMG data, is statistically sufficient to categorize motor impairment levels across a multitude of standard motor assessments in stroke patients.

In future research, this could enable low–bandwidth wearable recording devices for continuous monitoring, further simplifying the deployment of ML–driven applications in clinical environments [18,24]. Furthermore, we plan to validate the predictive model by comparing automated EMG–based predictions with therapist scoring, enabled by recruiting more patients to participate in such studies.

## Figures and Tables

**Table 1:** Patient demographic and clinical characteristics (*n* = 25).

Characteristic	Value
Demographics	
Age (years)	66.4 ± 12.1
Gender (male/female)	18 (72%)/7 (28%)
Lesion (CI/ICH)	13 (52%)/12 (48%)
Affected extremity (right/left)	12 (48%)/13 (52%)
Post–onset (days)	16.0 ± 8.6
Clinical scores	
FMA–UE (0 to 66)	37 ± 20
BRS Hand (1 to 6) * MAS Hand (MAS = 0/MAS > 0) **	4 ± 1
	16 (64%)/9 (36%)

CI—cerebral infarction; ICH—intracerebral hemorrhage; FMA–UE—the Fugl–Meyer Assessment for Upper Extremity. * Brunnstrom Recovery Stages (BRS) for the hand. ** Modified Ashworth Scale (MAS) for the hand, presented as a count (percentage) of patients with and without spasticity.

## Data Availability

In accordance with Japan’s Act on the Protection of Information (APPI) and directives from the Clinical Ethics Review Board of University Tsukuba Hospital, the collected data are not publicly accessible. For additional information, please consult H.K.

## Supplementary Materials

The following supporting information can be downloaded at: https://www.mdpi.com/article/10.3390/s25226829/s1, Figure S1: Confusion matrices of the best binary hand–gesture models using LDA and binary feature sets; Table S1: Performance metrics for a single gesture using LDA binary classification; Table S2: Performance metrics for the best binary gesture model combinations using LDA binary classification.
