# Supplementary material for: Automated EMG-Based Classification of Upper Extremity Motor Impairment Levels in Subacute Stroke
Source: Sensors (Basel). 2025 Nov 7;25(22):6829. doi: 10.3390/s25226829 (PMC12656085; doi:10.3390/s25226829)
Supplement: Supplementary file 1 [file sensors-25-06829-s001.zip › sensors-3930111-supplementary.pdf]

sensors

MDPI

Article

Automated EMG–Based Classification of Upper Extremity Motor Impairment Levels in Subacute Stroke

Alexey Anastasiev [1], Hideki Kadone [2]*, Aiki Marushima [3], Hiroki Watanabe [3], Alexander Zaboronok [3], Shinya Watanabe [3], Akira Matsumura [4], Kenji Suzuki [5], Yuji Matsumaru [3], Hiroyuki Nishiyama [6] and Eiichi Ishikawa [3]

[1] Department of Neurosurgery, University of Tsukuba Hospital, University of Tsukuba, 2-1-1 Amakubo, Tsukuba, 305-8576 Ibaraki, Japan; anastasiev.alexey.gb@u.tsukuba.ac.jp
[2] Center for Cybernics Research (CCR), Institute of Medicine, University of Tsukuba, 1-1-1 Tennodai, Tsukuba, 305-8575 Ibaraki, Japan
[3] Department of Neurosurgery, Institute of Medicine, University of Tsukuba, 1-1-1 Tennodai, Tsukuba, 305-8575 Ibaraki, Japan; aiki.marushima@md.tsukuba.ac.jp (A.M.); watanabe.hiroki.gb@u.tsukuba.ac.jp (H.W.); a.zaboronok@md.tsukuba.ac.jp (A.Z.); shinya-watanabey@md.tsukuba.ac.jp (S.W.); yujimatsumaru@md.tsukuba.ac.jp (Y.M.); e-ishikawa@md.tsukuba.ac.jp (E.I.)
[4] Ichihara Hospital, 3681 Ozone, Tsukuba, 300-3295 Ibaraki, Japan; matsumura.akira.ft@alumni.tsukuba.ac.jp
[5] Artificial Intelligence Laboratory, Center for Cybernics Research, Institute of Systems and Information Engineering, University of Tsukuba, 1-1-1 Tennodai, Tsukuba, 305-8573 Ibaraki, Japan; kenji@ieee.org
[6] Center for Cyber Medicine Research, University of Tsukuba, 1-1-1 Amakubo, Tsukuba, 305-8575 Ibaraki, Japan; nishiuro@md.tsukuba.ac.jp
* Correspondence: kadone@ccr.tsukuba.ac.jp

Supplementary materials:

Table S1. Performance metrics for a single gesture using LDA binary classification.

Metrics	Gesture	ACC	REC	PREC	SPEC	F1–Score	AUC–ROC	AUC–PRC
FMA–UE > 29	**Rest**	**0.878 ± 0.249**	**0.883 ± 0.285**	**0.880 ± 0.285**	**0.871 ± 0.325**	**0.873 ± 0.278**	**0.907 ± 0.241**	**0.937 ± 0.159**
	Hand fist	0.490 ± 0.310	0.480 ± 0.446	0.428 ± 0.410	0.509 ± 0.487	0.430 ± 0.391	0.442 ± 0.410	0.694 ± 0.224
	Index pinch	0.533 ± 0.309	0.517 ± 0.444	0.477 ± 0.419	0.553 ± 0.485	0.471 ± 0.394	0.546 ± 0.405	0.733 ± 0.231
	Flexion	0.532 ± 0.296	0.594 ± 0.442	0.490 ± 0.387	0.456 ± 0.488	0.510 ± 0.374	0.561 ± 0.403	0.733 ± 0.233
	Extension	0.527 ± 0.312	0.517 ± 0.448	0.466 ± 0.417	0.541 ± 0.489	0.465 ± 0.396	0.584 ± 0.419	0.757 ± 0.237
	Opening	0.815 ± 0.262	0.742 ± 0.389	0.791 ± 0.386	0.903 ± 0.288	0.752 ± 0.375	0.830 ± 0.303	0.892 ± 0.188
	Thumbs–up	0.372 ± 0.304	0.410 ± 0.438	0.333 ± 0.365	0.326 ± 0.457	0.348 ± 0.362	0.401 ± 0.417	0.674 ± 0.220
FMA–UE > 44	**Rest**	**0.922 ± 0.181**	**0.908 ± 0.280**	**0.879 ± 0.296**	**0.934 ± 0.222**	**0.886 ± 0.284**	**0.951 ± 0.155**	**0.944 ± 0.165**
	Hand fist	0.420 ± 0.299	0.383 ± 0.471	0.261 ± 0.352	0.451 ± 0.437	0.295 ± 0.372	0.375 ± 0.394	0.578 ± 0.232
	Index pinch	0.482 ± 0.316	0.425 ± 0.484	0.310 ± 0.387	0.521 ± 0.451	0.343 ± 0.403	0.455 ± 0.417	0.627 ± 0.259
	Flexion	0.570 ± 0.306	0.584 ± 0.481	0.426 ± 0.398	0.554 ± 0.442	0.473 ± 0.407	0.656 ± 0.378	0.718 ± 0.268
	Extension	0.568 ± 0.334	0.597 ± 0.481	0.451 ± 0.413	0.539 ± 0.450	0.494 ± 0.418	0.597 ± 0.406	0.696 ± 0.270
	Opening	0.456 ± 0.308	0.438 ± 0.484	0.300 ± 0.369	0.478 ± 0.455	0.352 ± 0.392	0.339 ± 0.387	0.623 ± 0.253
	Thumbs–up	0.374 ± 0.292	0.357 ± 0.466	0.228 ± 0.323	0.380 ± 0.426	0.266 ± 0.354	0.365 ± 0.406	0.587 ± 0.233
BRS > 3	**Rest**	**0.895 ± 0.124**	**0.955 ± 0.111**	**0.929 ± 0.085**	**0.583 ± 0.493**	**0.937 ± 0.081**	**0.940 ± 0.135**	**0.989 ± 0.026**
	Hand fist	0.659 ± 0.170	0.784 ± 0.204	0.795 ± 0.058	0.010 ± 0.099	0.780 ± 0.136	0.317 ± 0.222	0.843 ± 0.073
	Index pinch	0.759 ± 0.150	0.844 ± 0.169	0.872 ± 0.095	0.320 ± 0.466	0.847 ± 0.113	0.823 ± 0.199	0.968 ± 0.041
	Flexion	0.654 ± 0.155	0.768 ± 0.191	0.806 ± 0.071	0.060 ± 0.237	0.776 ± 0.128	0.411 ± 0.244	0.871 ± 0.073
	Extension	0.660 ± 0.156	0.778 ± 0.189	0.803 ± 0.077	0.045 ± 0.207	0.781 ± 0.128	0.239 ± 0.297	0.804 ± 0.098
	Opening	0.638 ± 0.173	0.760 ± 0.207	0.787 ± 0.073	0.005 ± 0.071	0.764 ± 0.144	0.256 ± 0.235	0.819 ± 0.083
	Thumbs–up	0.683 ± 0.170	0.807 ± 0.203	0.806 ± 0.066	0.037 ± 0.190	0.797 ± 0.137	0.324 ± 0.306	0.835 ± 0.096
BRS > 4	**Rest**	**0.806 ± 0.187**	**0.919 ± 0.171**	**0.853 ± 0.162**	**0.526 ± 0.499**	**0.870 ± 0.136**	**0.784 ± 0.307**	**0.930 ± 0.109**
	Hand fist	0.595 ± 0.222	0.733 ± 0.284	0.705 ± 0.224	0.257 ± 0.437	0.696 ± 0.216	0.554 ± 0.332	0.853 ± 0.128
	Index pinch	0.556 ± 0.230	0.700 ± 0.292	0.672 ± 0.228	0.200 ± 0.400	0.665 ± 0.226	0.332 ± 0.398	0.754 ± 0.161
	Flexion	0.550 ± 0.213	0.702 ± 0.299	0.664 ± 0.222	0.176 ± 0.381	0.659 ± 0.222	0.493 ± 0.362	0.826 ± 0.144
	Extension	0.488 ± 0.212	0.660 ± 0.306	0.597 ± 0.204	0.061 ± 0.240	0.613 ± 0.231	0.227 ± 0.273	0.720 ± 0.127
	Opening	0.480 ± 0.208	0.659 ± 0.298	0.594 ± 0.195	0.037 ± 0.189	0.611 ± 0.225	0.332 ± 0.324	0.764 ± 0.139
	Thumbs–up	0.514 ± 0.221	0.412 ± 0.336	0.797 ± 0.139	0.687 ± 0.316	0.615 ± 0.218	0.084 ± 0.278	0.632 ± 0.240
MAS > 0	**Rest**	**0.875 ± 0.205**	**0.886 ± 0.318**	**0.787 ± 0.347**	**0.868 ± 0.274**	**0.819 ± 0.325**	**0.951 ± 0.176**	**0.959 ± 0.139**

	Hand fist	0.599 ± 0.277	0.410 ± 0.492	0.305 ± 0.405	0.711 ± 0.358	0.337 ± 0.423	0.584 ± 0.429	0.699 ± 0.287
	Index pinch	0.371 ± 0.271	0.210 ± 0.407	0.116 ± 0.248	0.468 ± 0.389	0.144 ± 0.290	0.312 ± 0.379	0.519 ± 0.230
	Flexion	0.320 ± 0.258	0.153 ± 0.360	0.087 ± 0.228	0.421 ± 0.370	0.106 ± 0.261	0.188 ± 0.326	0.458 ± 0.193
	Extension	0.610 ± 0.272	0.501 ± 0.500	0.354 ± 0.399	0.679 ± 0.352	0.401 ± 0.420	0.587 ± 0.407	0.688 ± 0.279
	Opening	0.451 ± 0.278	0.384 ± 0.486	0.218 ± 0.313	0.490 ± 0.388	0.268 ± 0.356	0.364 ± 0.407	0.557 ± 0.254
	Thumbs–up	0.473 ± 0.298	0.461 ± 0.498	0.279 ± 0.347	0.482 ± 0.379	0.334 ± 0.382	0.414 ± 0.401	0.577 ± 0.254

FMA−UE – Fugl–Meyer Assessment–Upper Extremity, BRS – Brunnstrom stages, MAS – Modified Ashworth Scale, ACC – Accuracy, REC – Recall, PREC – Accuracy, SPEC – Recall, PREC – Precision, SPEC – Specificity, AUC–ROC – the area under the receiver operating characteristic curve, AUC–PRC – the area under the precision–recall curve. All metrics are shown with the standard deviation.

Table S2. Performance metrics for the best binary gesture model combinations using LDA binary classification.

Metrics	Gestures	ACC	REC	PREC	SPEC	F1–Score	AUC–ROC	AUC–PRC
FMA−UE > 29	Flexion and Thumbs–up	0.788 ± 0.260	0.737 ± 0.392	0.757 ± 0.391	0.856 ± 0.344	0.728 ± 0.374	0.897 ± 0.272	0.951 ± 0.137
FMA−UE > 44	Flexion and Thumbs–up	0.973 ± 0.104	0.970 ± 0.169	0.952 ± 0.190	0.976 ± 0.124	0.958 ± 0.178	0.981 ± 0.103	0.978 ± 0.102
BRS > 3	Rest and Hand fist	0.972 ± 0.097	0.976 ± 0.095	0.991 ± 0.042	0.953 ± 0.213	0.981 ± 0.072	0.968 ± 0.130	0.991 ± 0.038
BRS > 4	Pinch and Thumbs–up	0.966 ± 0.100	0.957 ± 0.132	0.993 ± 0.063	0.987 ± 0.113	0.970 ± 0.096	0.993 ± 0.068	0.997 ± 0.030
MAS > 0	Rest and Hand fist	0.970 ± 0.105	0.929 ± 0.257	0.924 ± 0.260	0.994 ± 0.055	0.926 ± 0.258	0.997 ± 0.041	0.997 ± 0.041

FMA−UE – Fugl–Meyer Assessment–Upper Extremity, BRS – Brunnstrom stages, MAS – Modified Ashworth Scale, ACC – Accuracy, REC – Recall, PREC – Accuracy, SPEC – Recall, PREC – Precision, SPEC – Specificity, AUC–ROC – the area under the receiver operating characteristic curve, AUC–PRC – the area under the precision–recall curve. All metrics are shown with the standard deviation.

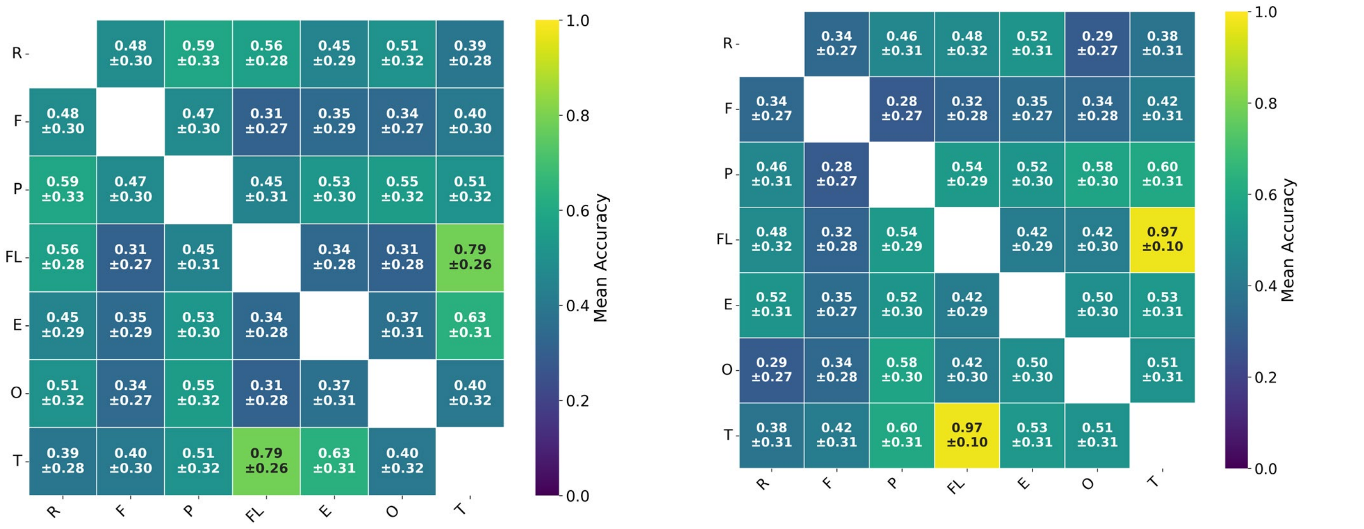

(**a**) (**b**)

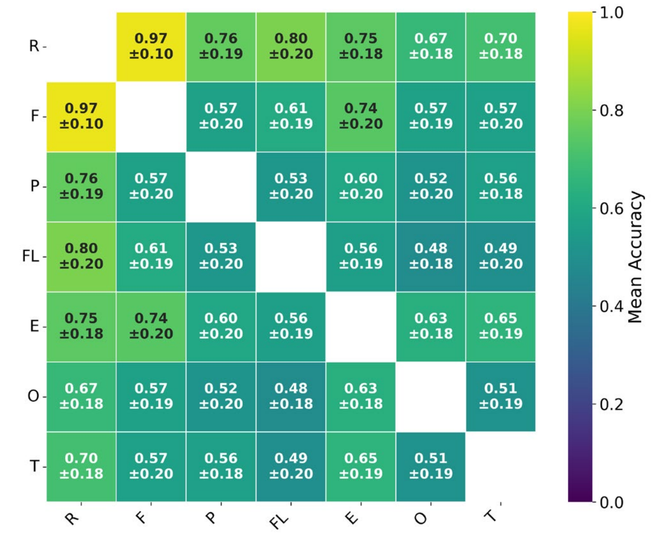

(**c**)

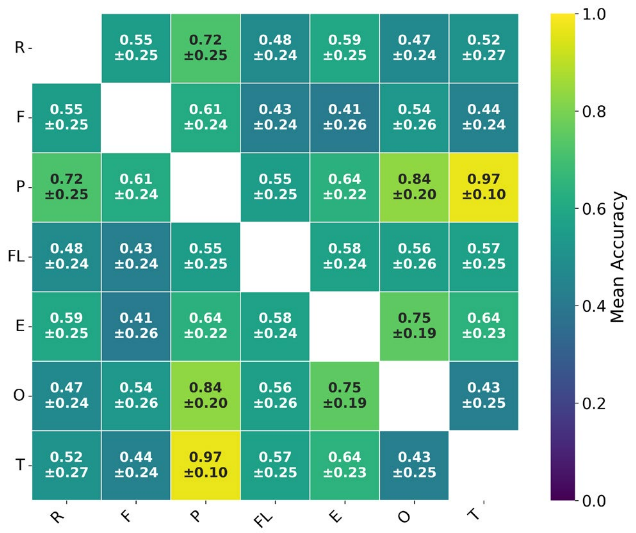

(**d**)

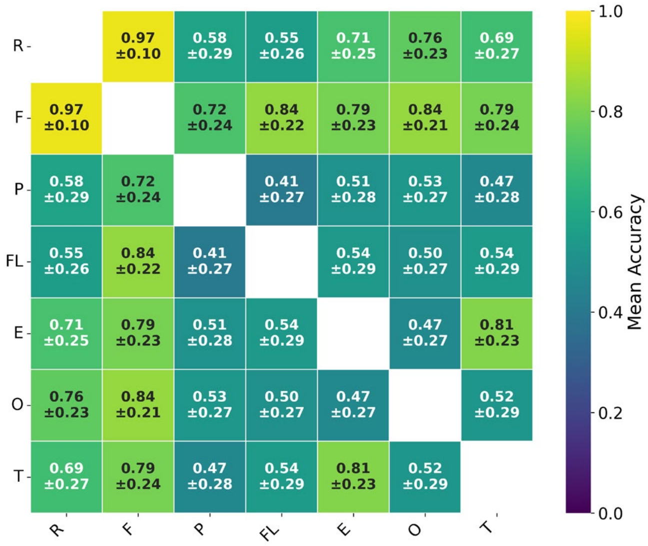

(**e**)

Figure S1. Confusion matrices of the best binary hand gesture models using LDA and binary feature sets: (**a**) FMA–UE > 29, (**b**) FMA–UE > 44, (**c**) BRS > 3, (**d**) BRS > 4, (**e**) MAS > 0; each for binary predictions across all possible gesture combinations (including rest).
